# Supplementary material for: The Eyes Absent family members EYA4 and EYA1 promote PLK1 activation and successful mitosis through tyrosine dephosphorylation
Source: Nat Commun. 2024 Feb 15;15:1385. doi: 10.1038/s41467-024-45683-4 (PMC10869800; doi:10.1038/s41467-024-45683-4)
Supplement: Supplementary file 1 — Supplementary Information [file 41467_2024_45683_MOESM1_ESM.pdf]

Supplementary Information

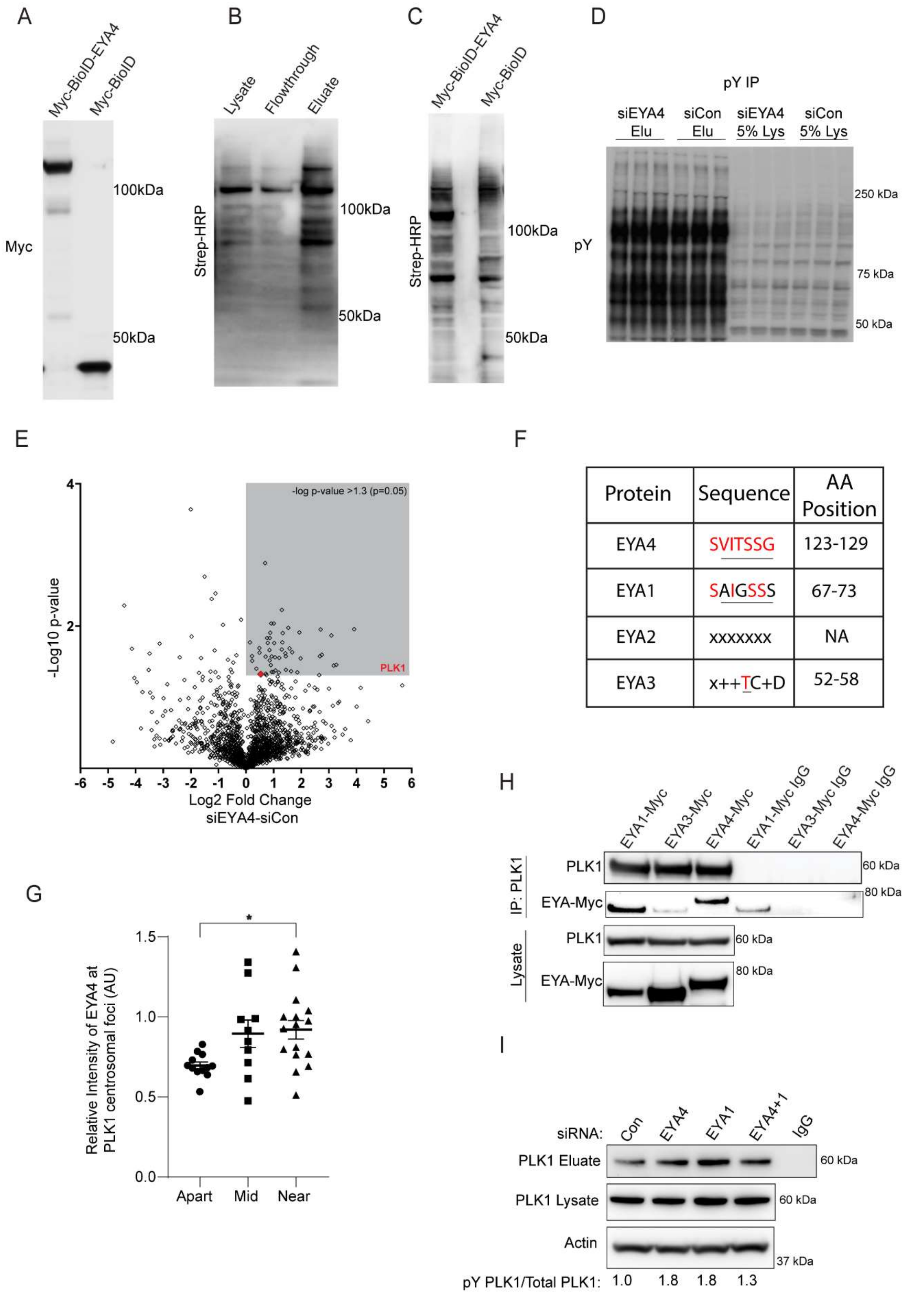

Supplementary Figure 1| EYA4 and EYA1 interact with and dephosphorylate PLK1. a, Western blot from 293T cells expressing EYA4-Myc-BioID and Myc-BioID constructs. **b,** Western blot showing

enrichment of biotinylated proteins following precipitation with streptavidin. **c,** Western blot showing equal levels of biotinylated proteins in samples destined for trypsin digestion and mass spectrometry. **d,** Immunoprecipitation and western blotting of tyrosine phosphorylated proteins using an anti-phosphotyrosine antibody following depletion of EYA4 showing IP lysates (Lys) and Eluates (Elu). **e,** Volcano plot of immunoprecipitated tyrosine phosphorylated proteins identified by mass spectrometry, proteins with increased tyrosine phosphorylation following EYA4 depletion are on the right side (n=2). Grey coloured box represents 50 proteins with statistically significant increases in tyrosine phosphorylation (-log p-value > 1.3). PLK1 is indicated on the plot. **f,** Table showing conserved putative PDS and phosphosite in EYA4 and EYA1, but not present in EYA2 or EYA3. Red letters are conserved within the EYA4 sequence, underlined sequences match the PLK1 consensus PDS, x indicates amino acid not present, + indicates extra amino acids prior to alignments (all alignments performed using the NCBI protein blast tool) **g,** Analysis of EYA4 intensity at PLK1 centrosomal foci in G2 arrested cells with different positional conformations in association with Figure 1G (n = 38 cells, only cells with two centrosomes were used for analysis, * $p \leq 0.05$). **h,** PLK1 coimmunopurification with Myc-tagged EYA4, and EYA1, but not EYA3, in G2 arrested cells. **i,** Immunoprecipitation of tyrosine phosphorylated proteins using an anti pY antibody and western blotting for PLK1. Densitometry values of PLK1 in the eluate (pY PLK1) relative to total PLK1 in the lysate is under the blot. Source data for is provided as a Source data file.

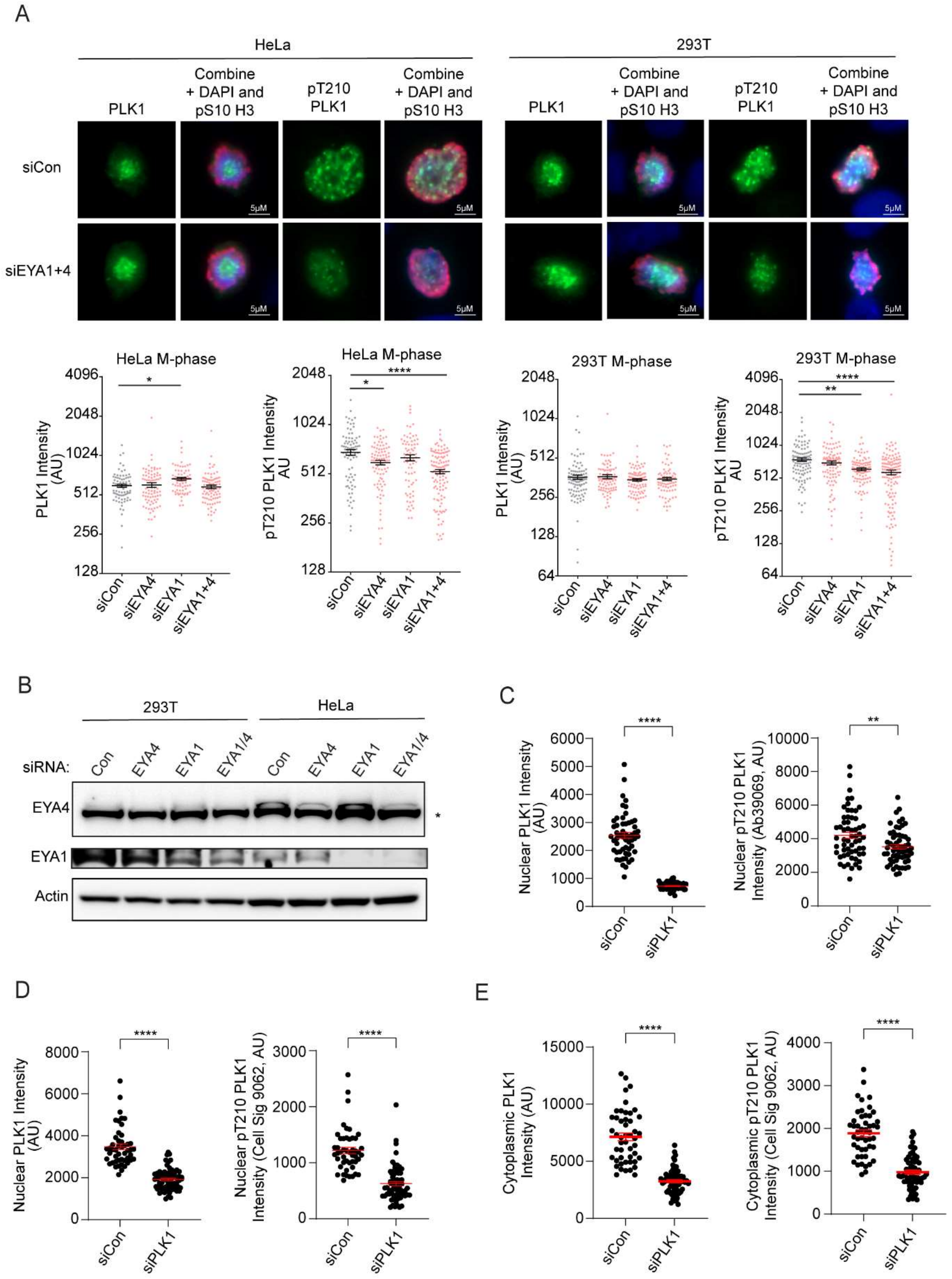

Supplementary Figure 2| Mitotic pT210 staining, EYA knockdown validations, and pT210 antibody validations. **a,** Representative immunofluorescence images of mitotic HeLa and 293T cells stained for PLK1 or pT210 (Ab39069), alone and in combination with the mitotic marker pS10H3 and DAPI (top panel). Fluorescence intensity quantitation of nuclear PLK1 or pT210 (n ≥ 74 mitotic cells * p ≤ 0.05, ** p ≤ 0.01, **** p ≤ 0.0001) (bottom panel). **b,** Western blots confirming EYA4, EYA1 and combination knockdowns in asynchronous 293T and HeLa cells. The asterisk (*) represents a non-specific band on

the EYA4 blot. **c,** Mitotic nuclear total PLK1 and pT210 PLK1 (Ab39069) intensity from fluorescent imaging validation experiments including siCon and siPLK1 treatments (n ≥ 55 mitotic cells, ** $p \leq 0.01$, **** $p \leq 0.0001$). **d,** Mitotic nuclear total PLK1 and pT210 PLK1 (Cell Signalling 9062) from fluorescent imaging validation experiment including siCon and siPLK1 treatments (n ≥ 48 mitotic cells, **** ≤ 0.0001). **e,** Mitotic cytoplasmic total PLK1 and pT210 PLK1 (Cell Signalling 9062) from fluorescent imaging validation experiment including siCon and siPLK1 treatments (n ≥ 48 mitotic cells, **** ≤ 0.0001). Source data is provided as a Source data file.

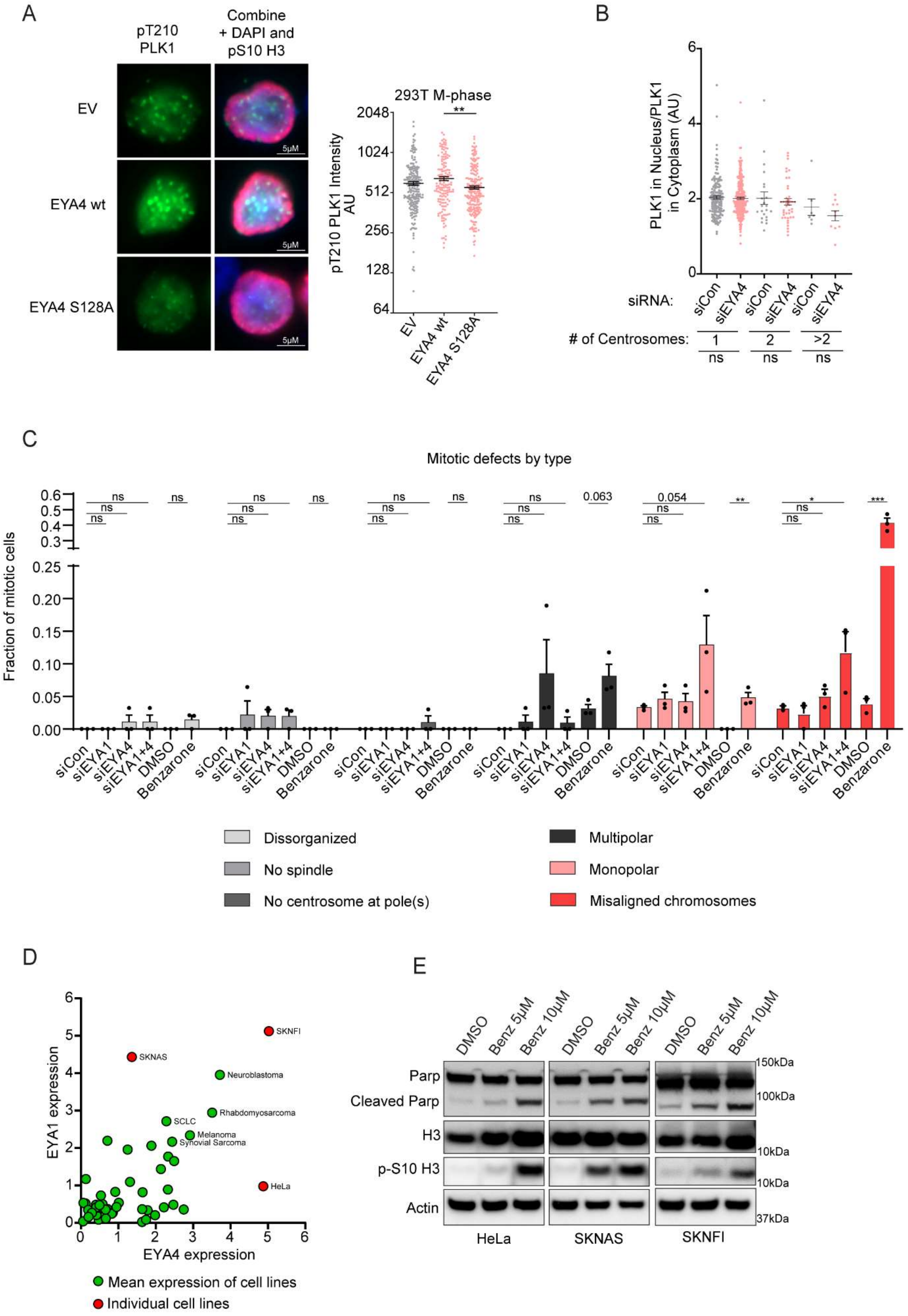

Supplementary Figure 3| Effects of the EYAs on pT210 phosphorylation, mitotic aberrations, mitotic index and cell death. a, Representative immunofluorescence images of mitotic 293T cells overexpressing EYA4 WT or an S128A mutant stained for pT210 (Ab39069), alone and in combination

with the mitotic marker pS10H3 and DAPI (left panel). Fluorescence intensity quantitation of nuclear pT210 ($n \geq 150$ mitotic cells ** $p \leq 0.01$) (right panel). **b,** Immunofluorescence quantitation related to Figure 3D-F. The ratio of PLK1 in the nucleus relative to that in the cytoplasm is presented in G2 cells with variable numbers of centrosomes ($n \geq 164$ cells with one centrosome focus, ≥ 22 cells with two centrosome foci, and ≥ 7 cells with more than two centrosomal foci). **c,** Spindle defect subdivision by type related to figure 3G-H. Co-depletion of EYA4 and EYA1 induced a moderate number of monopolar spindles as well as spindles with misaligned chromosomes ($n \geq 87$ mitotic cells across three experiments, * $p \leq 0.05$, ** $p \leq 0.01$, *** $p \leq 0.001$). **d,** Public gene expression data for EYA1 and EYA4 (DepMap Portal) presented either as the mean across tumour types (green circles), or values for individual cell lines (HeLa, SKNAS, SKNFI, red circles). **e,** Western blots of cleaved PARP and pS10 H3 were performed in response to treatment with benzarone in cell lines with high levels of EYA1 (SKNAS), high levels of EYA4 (HeLa), or both (SKNFI). Source data is provided as a Source data file.

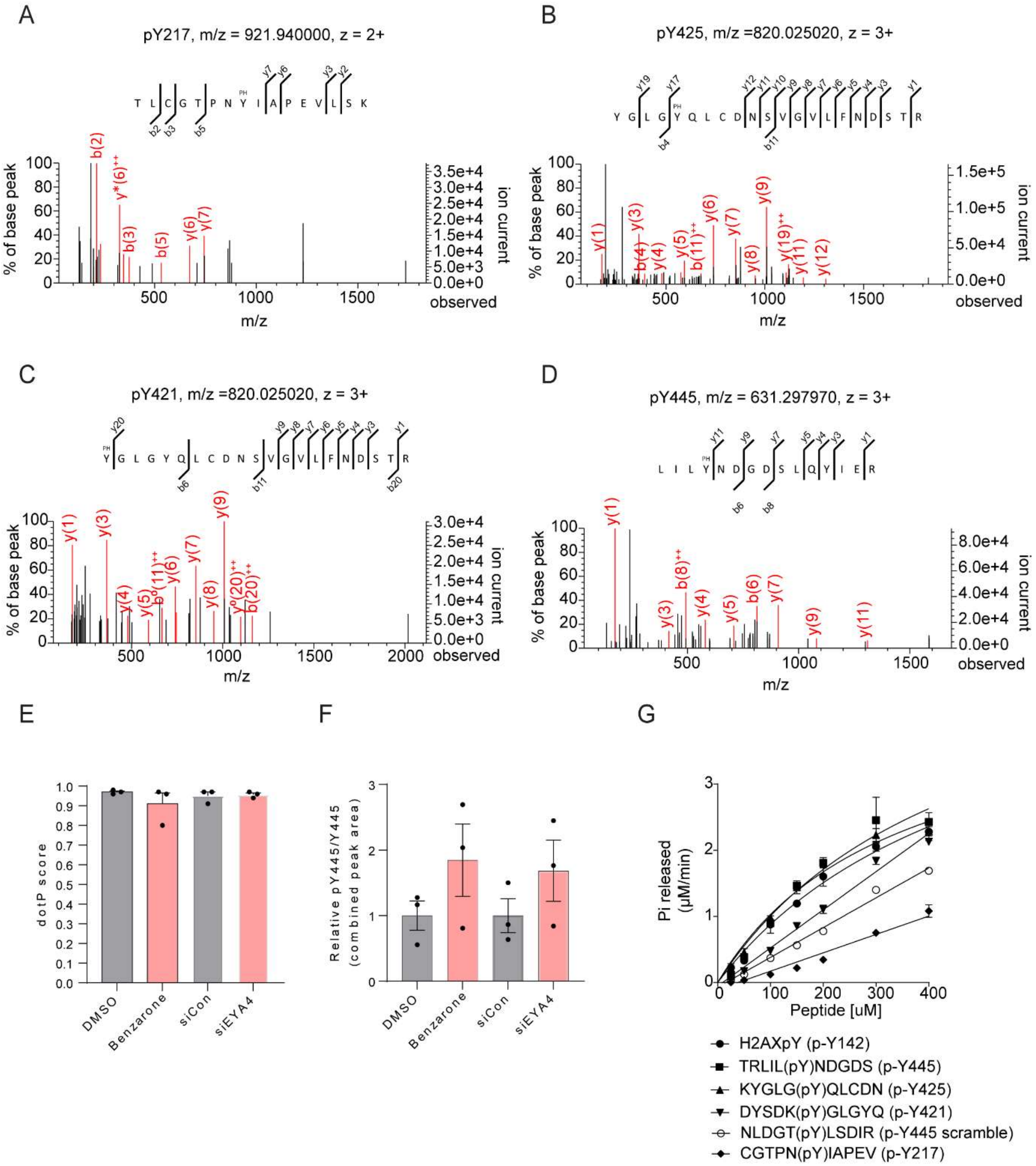

Supplementary Figure 4| PLK1 tyrosine phosphosites and their dephosphorylation. a-d, Representative mass spectra from PRM mass spec experiments corresponding to pY217 **(a),** pY425 **(b),** pY421 **(c),** and pY445 **(d)**. Peptides with designated y and b fragment ions are shown above each spectra. PH above Y indicates phosphorylation. **e,** pY445 peptide dot product scores from PRM data following a two-hour treatment with benzarone (10µM), or depletion of EYA4 (n=3). **f,** Relative pY445/Y445 peptide combined peak area from 6 fragment ions (n=3). **g,** *In-vitro* phosphatase assay data from figure 4C presented on a single plot. Source data is provided as a Source data file.

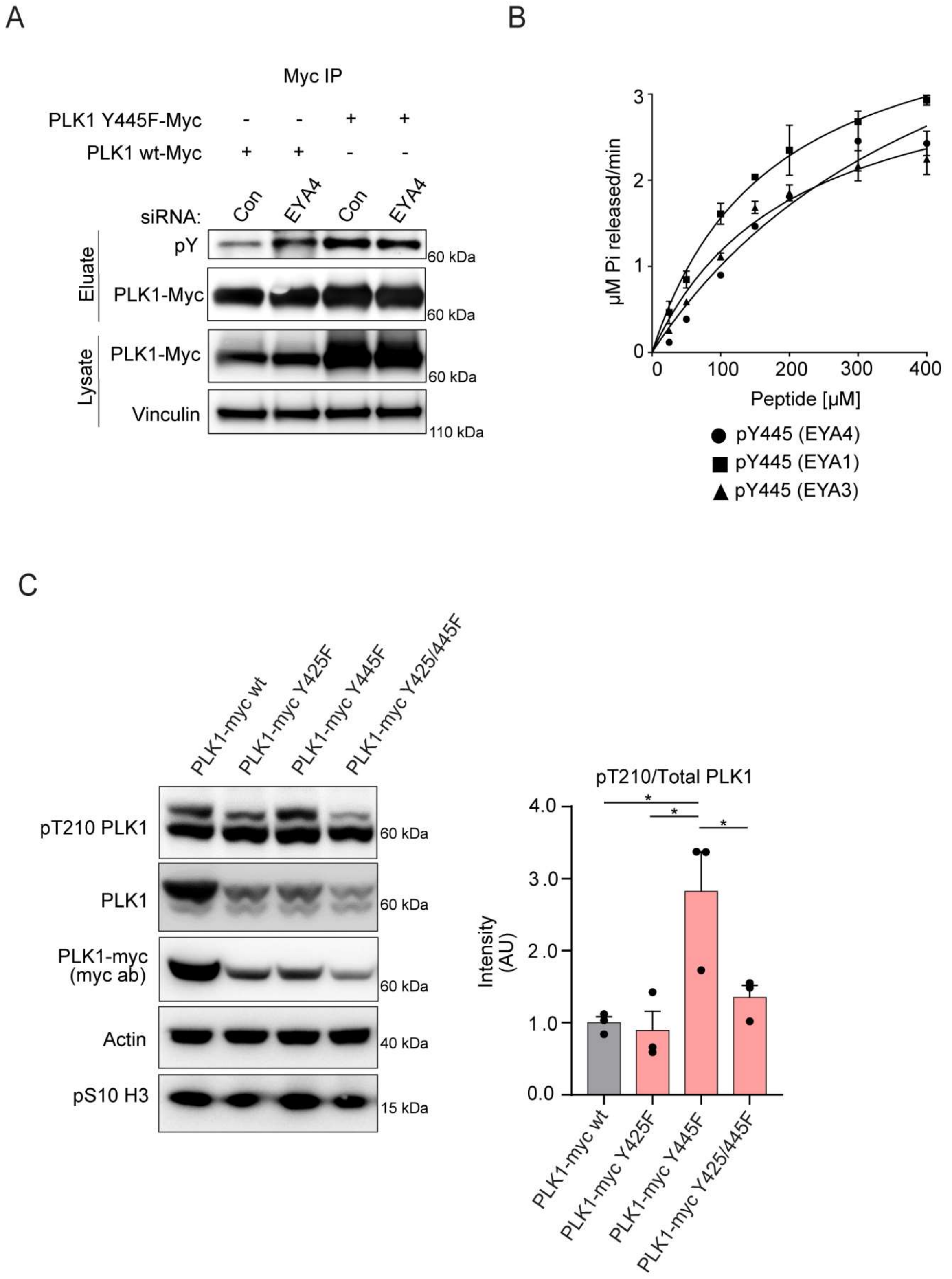

Supplementary Figure 5| PLK1 phosphomutant analysis and dephosphorylation of pY445 by phosphatase domains of the EYAs. a, Immunoblotting of total tyrosine phosphorylation on immunoprecipitated PLK1 or a Y445F mutant in cells arrested in G2 with RO3306 following treatment with a control siRNA or one targeting EYA4. **b,** *In-vitro* phosphatase assays using the phosphatase domains of EYA4, EYA1, and EYA3 and a pY445 phosphopeptide. All dephosphorylation reactions fit with Michaelis-Menten kinetics, error bars represent standard deviations. **c,** Expression of myc-tagged WT PLK1 or phosphosite mutants including Y425F, Y445F and a combination mutant in nocodazole

arrested mitotic 293T cells. Densitometry of the T210 PLK1/total PLK1 ratio is presented (n=3, * $p \leq 0.05$). Source data is provided as a Source data file.

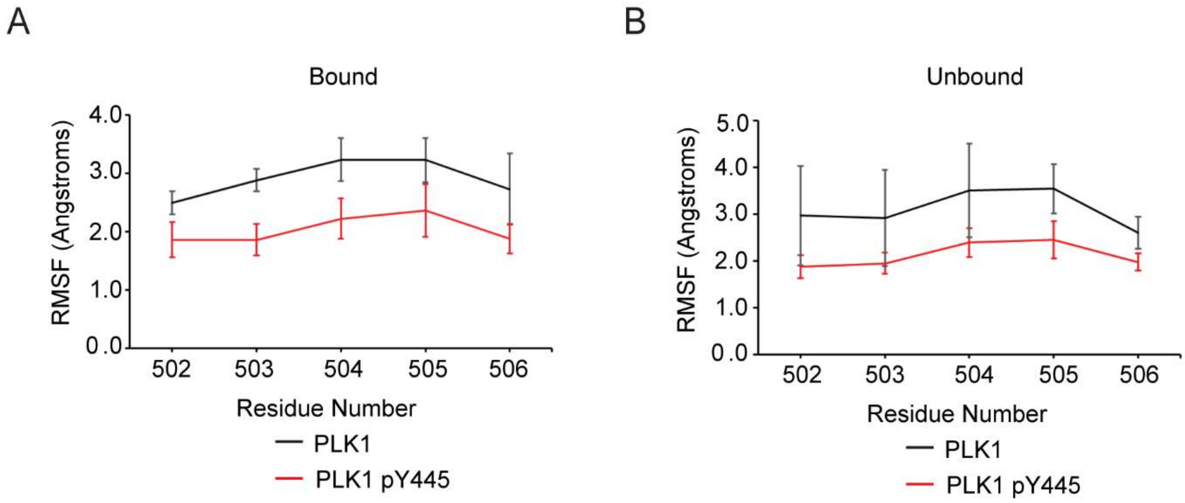

Supplementary Figure 6| RMSF plots of PLK1 residues 502-506. a-b, Close-up plots of residues 502-506 of the WT PLK1 PBD structure (black line), or pY445 simulated PLK1 PBD structure (red line). Plots are either bound to a model phosphopeptide **(a)**, or unbound **(b)** (n= 5 simulations). Error bars represent standard deviations. Source data is provided as a Source data file.
