# Supplementary material for: The Eyes Absent family members EYA4 and EYA1 promote PLK1 activation and successful mitosis through tyrosine dephosphorylation
Source: Nat Commun. 2024 Feb 15;15:1385. doi: 10.1038/s41467-024-45683-4 (PMC10869800; doi:10.1038/s41467-024-45683-4)
Supplement: Supplementary file 3 — Description of Additional Supplementary Files [file 41467_2024_45683_MOESM3_ESM.pdf]

Description of Additional Supplementary Files

File Name: Supplementary Data 1
Description: Additional data including raw data related to BioID, and pY IP-MS experiments.

File Name: Supplementary Data 2
Description: Peptide information for PRM inclusion list and MM-GBSA calculations.
.
