# Supplementary material for: The Eyes Absent family members EYA4 and EYA1 promote PLK1 activation and successful mitosis through tyrosine dephosphorylation
Source: Nat Commun. 2024 Feb 15;15:1385. doi: 10.1038/s41467-024-45683-4 (PMC10869800; doi:10.1038/s41467-024-45683-4)
Supplement: Supplementary file 6 — Reporting Summary [file 41467_2024_45683_MOESM6_ESM.pdf]

nature portfolio

Corresponding author(s): Hilda Pickett

Last updated by author(s): 19/1/24

# Reporting Summary

Nature Portfolio wishes to improve the reproducibility of the work that we publish. This form provides structure for consistency and transparency in reporting. For further information on Nature Portfolio policies, see our Editorial Policies and the Editorial Policy Checklist.

Please do not complete any field with "not applicable" or n/a. Refer to the help text for what text to use if an item is not relevant to your study.
For final submission: please carefully check your responses for accuracy; you will not be able to make changes later.

## Statistics

For all statistical analyses, confirm that the following items are present in the figure legend, table legend, main text, or Methods section.

| n/a | Confirmed | |
|---|---|---|
| ☐ | ■ | The exact sample size (*n*) for each experimental group/condition, given as a discrete number and unit of measurement |
| ☐ | ■ | A statement on whether measurements were taken from distinct samples or whether the same sample was measured repeatedly |
| ☐ | ■ | The statistical test(s) used AND whether they are one- or two-sided<br>*Only common tests should be described solely by name; describe more complex techniques in the Methods section.* |
| x | ☐ | A description of all covariates tested |
| x | ☐ | A description of any assumptions or corrections, such as tests of normality and adjustment for multiple comparisons |
| ☐ | ■ | A full description of the statistical parameters including central tendency (e.g. means) or other basic estimates (e.g. regression coefficient) AND variation (e.g. standard deviation) or associated estimates of uncertainty (e.g. confidence intervals) |
| ☐ | ■ | For null hypothesis testing, the test statistic (e.g. *F*, *t*, *r*) with confidence intervals, effect sizes, degrees of freedom and *P* value noted<br>*Give P values as exact values whenever suitable.* |
| x | ☐ | For Bayesian analysis, information on the choice of priors and Markov chain Monte Carlo settings |
| x | ☐ | For hierarchical and complex designs, identification of the appropriate level for tests and full reporting of outcomes |
| x | ☐ | Estimates of effect sizes (e.g. Cohen's *d*, Pearson's *r*), indicating how they were calculated |

*Our web collection on statistics for biologists contains articles on many of the points above.*

## Software and code

Policy information about availability of computer code

| | |
|---|---|
| Data collection | Microsoft Excel, Cellprofiler v2 and v4 |
| Data analysis | Cellprofiler v2 and v4, Maxquant v1, Perseus v2, Proteome Discoverer, Skyline v19, Graphpad Prizm v7-10 |

For manuscripts utilizing custom algorithms or software that are central to the research but not yet described in published literature, software must be made available to editors and reviewers. We strongly encourage code deposition in a community repository (e.g. GitHub). See the Nature Portfolio guidelines for submitting code & software for further information.

## Data

Policy information about availability of data

All manuscripts must include a data availability statement. This statement should provide the following information, where applicable:

- Accession codes, unique identifiers, or web links for publicly available datasets
- A description of any restrictions on data availability
- For clinical datasets or third party data, please ensure that the statement adheres to our policy

All relevant data supporting the findings of this study are provided in the main figures and Supplementary Information files including raw data. The MD simulation input files, initial and final coordinate files have been deposited in Zenodo with the DOI, 10.5281/zenodo.10223834. The mass spectrom

# Research involving human participants, their data, or biological material

Policy information about studies with human participants or human data. See also policy information about sex, gender (identity/presentation), and sexual orientation and race, ethnicity and racism.

| | |
|---|---|
| Reporting on sex and gender | N/A |
| Reporting on race, ethnicity, or other socially relevant groupings | N/A |
| Population characteristics | N/A |
| Recruitment | N/A |
| Ethics oversight | N/A |

Note that full information on the approval of the study protocol must also be provided in the manuscript.

# Field-specific reporting

Please select the one below that is the best fit for your research. If you are not sure, read the appropriate sections before making your selection.

- [x] Life sciences
- [ ] Behavioural & social sciences
- [ ] Ecological, evolutionary & environmental sciences

For a reference copy of the document with all sections, see nature.com/documents/nr-reporting-summary-flat.pdf

# Life sciences study design

All studies must disclose on these points even when the disclosure is negative.

| | |
|---|---|
| Sample size | Sample sizes were chosen based on standards of the field. For example, for statistical consideration quantification of western blotting experiments typically n=3-4 (we did both), BioID proximity proteomics experiments are typically 3-5 (we used n=4), and mo |
| Data exclusions | No data were excluded |
| Replication | Sample sizes given in the manuscript and listed under "Sample size" represent biological replicates. In some cases additional biological replicates were performed and not included in the manuscript (i.e., knockdown validations were performed routinely by w |
| Randomization | Randomization was not required as the experiments in the manuscript all involved genetic or pharmacological manipulation of non-clonal cell lines (i.e. variability from the cell population is equally present in all samples). |
| Blinding | Image analysis was performed using cellprofiler and was therefore blind in all cases except 2A-C, 2G-H, Supp 1G, and Supp 3C. In these experiments masking of the cellular objects to be quantified were either unsatisfactory in cellprofiler, and were thus ma |

# Behavioural & social sciences study design

All studies must disclose on these points even when the disclosure is negative.

| | |
|---|---|
| Study description | N/A |
| Research sample | N/A |
| Sampling strategy | N/A |
| Data collection | N/A |
| Timing | N/A |
| Data exclusions | N/A |
| Non-participation | N/A |
| Randomization | N/A |

# Ecological, evolutionary & environmental sciences study design

All studies must disclose on these points even when the disclosure is negative.

| | |
|---|---|
| Study description | N/A |
| Research sample | N/A |
| Sampling strategy | N/A |
| Data collection | N/A |
| Timing and spatial scale | N/A |
| Data exclusions | N/A |
| Reproducibility | N/A |
| Randomization | N/A |
| Blinding | N/A |

Did the study involve field work? ☐ Yes ■ No

## Field work, collection and transport

| | |
|---|---|
| Field conditions | N/A |
| Location | N/A |
| Access & import/export | N/A |
| Disturbance | N/A |

# Reporting for specific materials, systems and methods

We require information from authors about some types of materials, experimental systems and methods used in many studies. Here, indicate whether each material, system or method listed is relevant to your study. If you are not sure if a list item applies to your research, read the appropriate section before selecting a response.

## Materials & experimental systems

| n/a | Involved in the study | |
|---|---|---|
| ☐ | ■ | Antibodies |
| ☐ | ■ | Eukaryotic cell lines |
| x | ☐ | Palaeontology and archaeology |
| x | ☐ | Animals and other organisms |
| x | ☐ | Clinical data |
| x | ☐ | Dual use research of concern |
| x | ☐ | Plants |

## Methods

| n/a | Involved in the study | |
|---|---|---|
| x | ☐ | ChIP-seq |
| x | ☐ | Flow cytometry |
| x | ☐ | MRI-based neuroimaging |

## Antibodies

| | |
|---|---|
| Antibodies used | The following antibodies were used in the manuscript: PLK_F-8 SC17783:_Santa Cruz (1:400), Phospho tyrosine_8954:_Cell Signaling (1:1000), EYA4_ab93865:_Abcam (1:1000), Actin _a2066:_Sigma (1:1000), Myc_9B11:_Cell Signaling ( |
| Validation | Validation of antibodies that lack prior publication of validation (EYA1, EYA4) was performed by siRNA kd, and in the case of EYA4, independently by KO. Additionally, pT210 PLK1 antibody validations were performed in IF experiments and t |

# Eukaryotic cell lines

Policy information about cell lines and Sex and Gender in Research

| | |
|---|---|
| Cell line source(s) | HeLa were obtained from the American Type Culture Collection. HEK293T were obtained from the Francis Crick Institute. SKNAS cells are from ATCC. SKNFI cells are from Dr. Loretta Lau. |
| Authentication | Cell lines were authenticated by 16-locus short-tandem-repeat profiling by CellBank Australia (Children's Medical Research Institute). |
| Mycoplasma contamination | Cell lines were tested for mycoplasma contamination by CellBank Australia (Children's Medical Research Institute). |
| Commonly misidentified lines (See ICLAC register) | No commonly misidentified cell lines were used. |

# Palaeontology and Archaeology

| | |
|---|---|
| Specimen provenance | N/A |
| Specimen deposition | N/A |
| Dating methods | N/A |

☐ Tick this box to confirm that the raw and calibrated dates are available in the paper or in Supplementary Information.

| | |
|---|---|
| Ethics oversight | N/A |

Note that full information on the approval of the study protocol must also be provided in the manuscript.

# Animals and other research organisms

Policy information about studies involving animals; ARRIVE guidelines recommended for reporting animal research, and Sex and Gender in Research

| | |
|---|---|
| Laboratory animals | N/A |
| Wild animals | N/A |
| Reporting on sex | N/A |
| Field-collected samples | N/A |
| Ethics oversight | N/A |

Note that full information on the approval of the study protocol must also be provided in the manuscript.

# Clinical data

Policy information about clinical studies

All manuscripts should comply with the ICMJE guidelines for publication of clinical research and a completed CONSORT checklist must be included with all submissions.

| | |
|---|---|
| Clinical trial registration | N/A |
| Study protocol | N/A |
| Data collection | N/A |
| Outcomes | N/A |

# Dual use research of concern

Policy information about dual use research of concern

## Hazards

Could the accidental, deliberate or reckless misuse of agents or technologies generated in the work, or the application of information presented in the manuscript, pose a threat to:

| No | Yes | |
|---|---|---|
| [x] | [ ] | Public health |
| [x] | [ ] | National security |
| [x] | [ ] | Crops and/or livestock |
| [x] | [ ] | Ecosystems |
| [x] | [ ] | Any other significant area |

### Experiments of concern

Does the work involve any of these experiments of concern:

| No | Yes | |
|---|---|---|
| [x] | [ ] | Demonstrate how to render a vaccine ineffective |
| [x] | [ ] | Confer resistance to therapeutically useful antibiotics or antiviral agents |
| [x] | [ ] | Enhance the virulence of a pathogen or render a nonpathogen virulent |
| [x] | [ ] | Increase transmissibility of a pathogen |
| [x] | [ ] | Alter the host range of a pathogen |
| [x] | [ ] | Enable evasion of diagnostic/detection modalities |
| [x] | [ ] | Enable the weaponization of a biological agent or toxin |
| [x] | [ ] | Any other potentially harmful combination of experiments and agents |

## Plants

| | |
|---|---|
| Seed stocks | N/A |
| Novel plant genotypes | N/A |
| Authentication | N/A |

## ChIP-seq

### Data deposition

[ ] Confirm that both raw and final processed data have been deposited in a public database such as GEO.

[ ] Confirm that you have deposited or provided access to graph files (e.g. BED files) for the called peaks.

| | |
|---|---|
| Data access links<br>*May remain private before publication.* | N/A |
| Files in database submission | N/A |
| Genome browser session<br>(e.g. UCSC) | N/A |

### Methodology

| | |
|---|---|
| Replicates | N/A |
| Sequencing depth | N/A |
| Antibodies | N/A |
| Peak calling parameters | N/A |
| Data quality | N/A |

Software N/A

## Flow Cytometry

### Plots

Confirm that:

- [ ] The axis labels state the marker and fluorochrome used (e.g. CD4-FITC).
- [ ] The axis scales are clearly visible. Include numbers along axes only for bottom left plot of group (a 'group' is an analysis of identical markers).
- [ ] All plots are contour plots with outliers or pseudocolor plots.
- [ ] A numerical value for number of cells or percentage (with statistics) is provided.

### Methodology

| | |
|---|---|
| Sample preparation | N/A |
| Instrument | N/A |
| Software | N/A |
| Cell population abundance | N/A |
| Gating strategy | N/A |

- [ ] Tick this box to confirm that a figure exemplifying the gating strategy is provided in the Supplementary Information.

## Magnetic resonance imaging

### Experimental design

| | |
|---|---|
| Design type | N/A |
| Design specifications | N/A |
| Behavioral performance measures | N/A |

| | |
|---|---|
| Imaging type(s) | N/A |
| Field strength | N/A |
| Sequence & imaging parameters | N/A |
| Area of acquisition | N/A |

Diffusion MRI ☐ Used ☐ Not used

### Preprocessing

| | |
|---|---|
| Preprocessing software | N/A |
| Normalization | N/A |
| Normalization template | N/A |
| Noise and artifact removal | N/A |
| Volume censoring | N/A |

### Statistical modeling & inference

| | |
|---|---|
| Model type and settings | N/A |
| Effect(s) tested | N/A |

Specify type of analysis: ☐ Whole brain ☐ ROI-based ☐ Both

Statistic type for inference (See Eklund et al. 2016) | N/A

Correction | N/A

## Models & analysis

| n/a | Involved in the study |
|---|---|
| x | ☐ Functional and/or effective connectivity |
| ☐ | ■ Graph analysis |
| x | ☐ Multivariate modeling or predictive analysis |

Functional and/or effective connectivity | N/A

Graph analysis | Graphs were made in graphpad prism.

Multivariate modeling and predictive analysis | N/A

This checklist template is licensed under a Creative Commons Attribution 4.0 International License, which permits use, sharing, adaptation, distribution and reproduction in any medium or format, as long as you give appropriate credit to the original author(s) and the source, provide a link to the Creative Commons license, and indicate if changes were made. The images or other third party material in this article are included in the article's Creative Commons license, unless indicated otherwise in a credit line to the material. If material is not included in the article's Creative Commons license and your intended use is not permitted by statutory regulation or exceeds the permitted use, you will need to obtain permission directly from the copyright holder. To view a copy of this license, visit http://creativecommons.org/licenses/by/4.0/
