# Supplementary material for: The Eyes Absent family members EYA4 and EYA1 promote PLK1 activation and successful mitosis through tyrosine dephosphorylation
Source: Nat Commun. 2024 Feb 15;15:1385. doi: 10.1038/s41467-024-45683-4 (PMC10869800; doi:10.1038/s41467-024-45683-4)

**Source data**

Uncropped Western Blots

Figure 1 and Supplementary Figure 1:


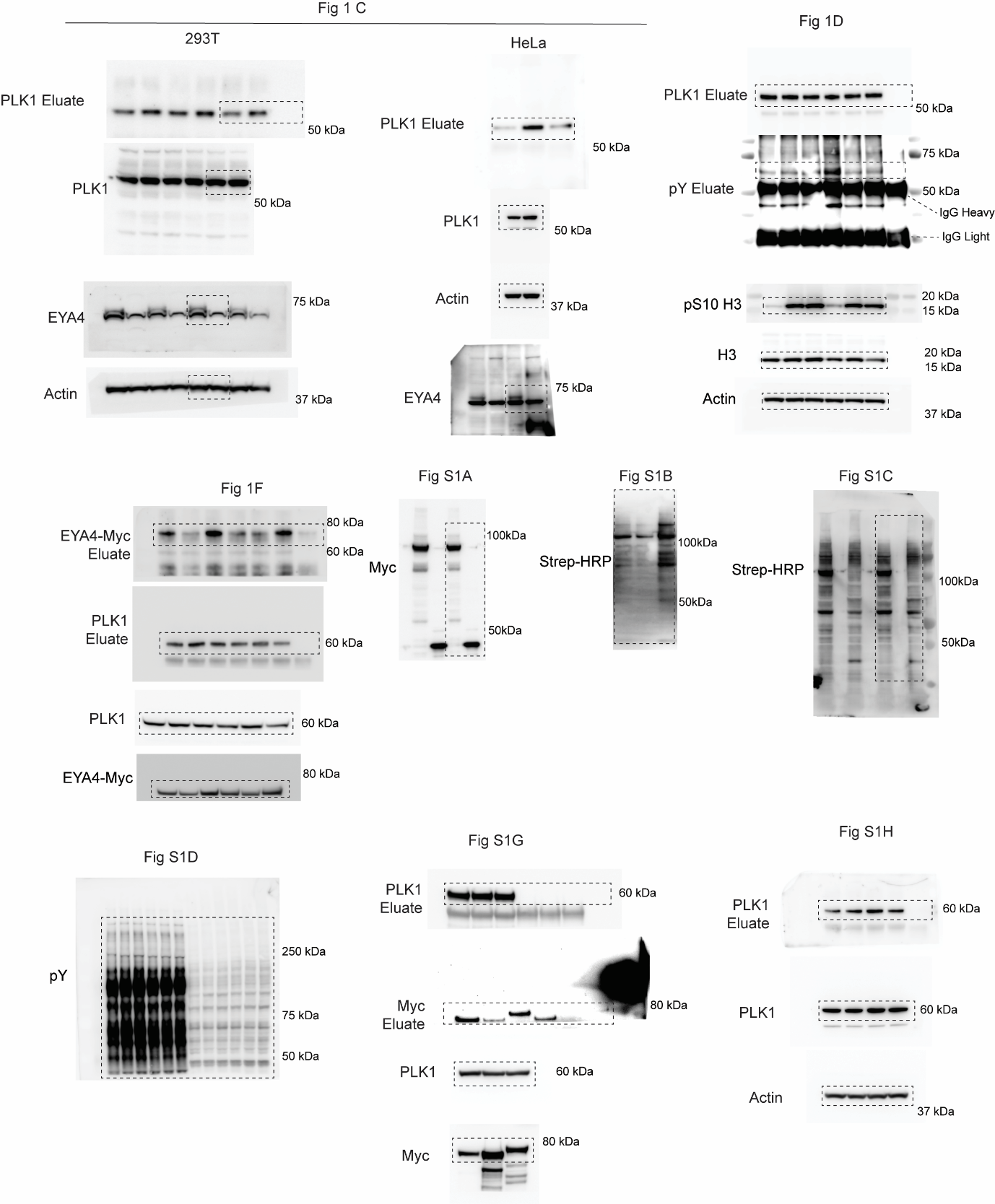


Figure 2 and Supplementary Figure 2:


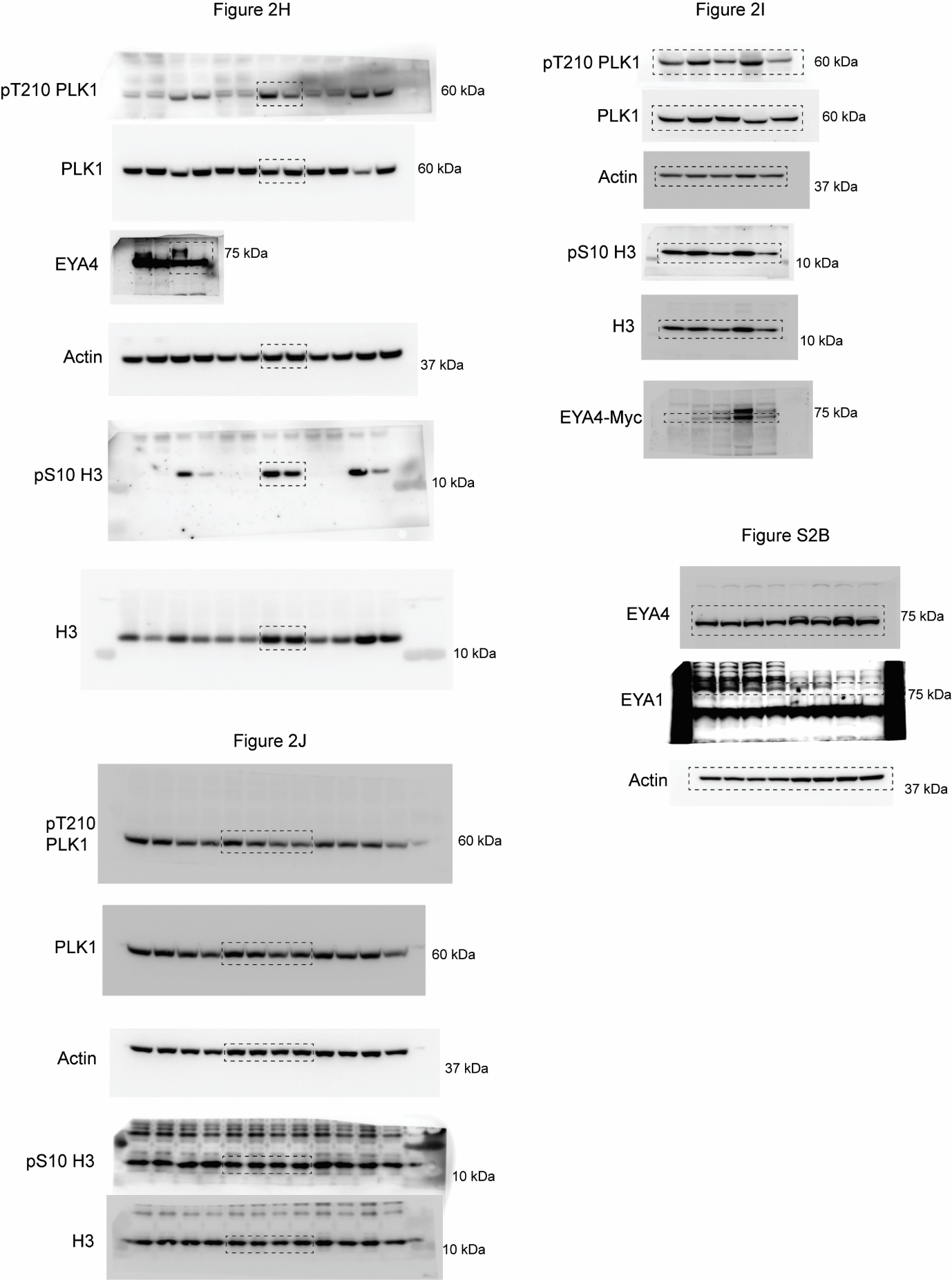


Figure 4 and Supplementary Figure 3:


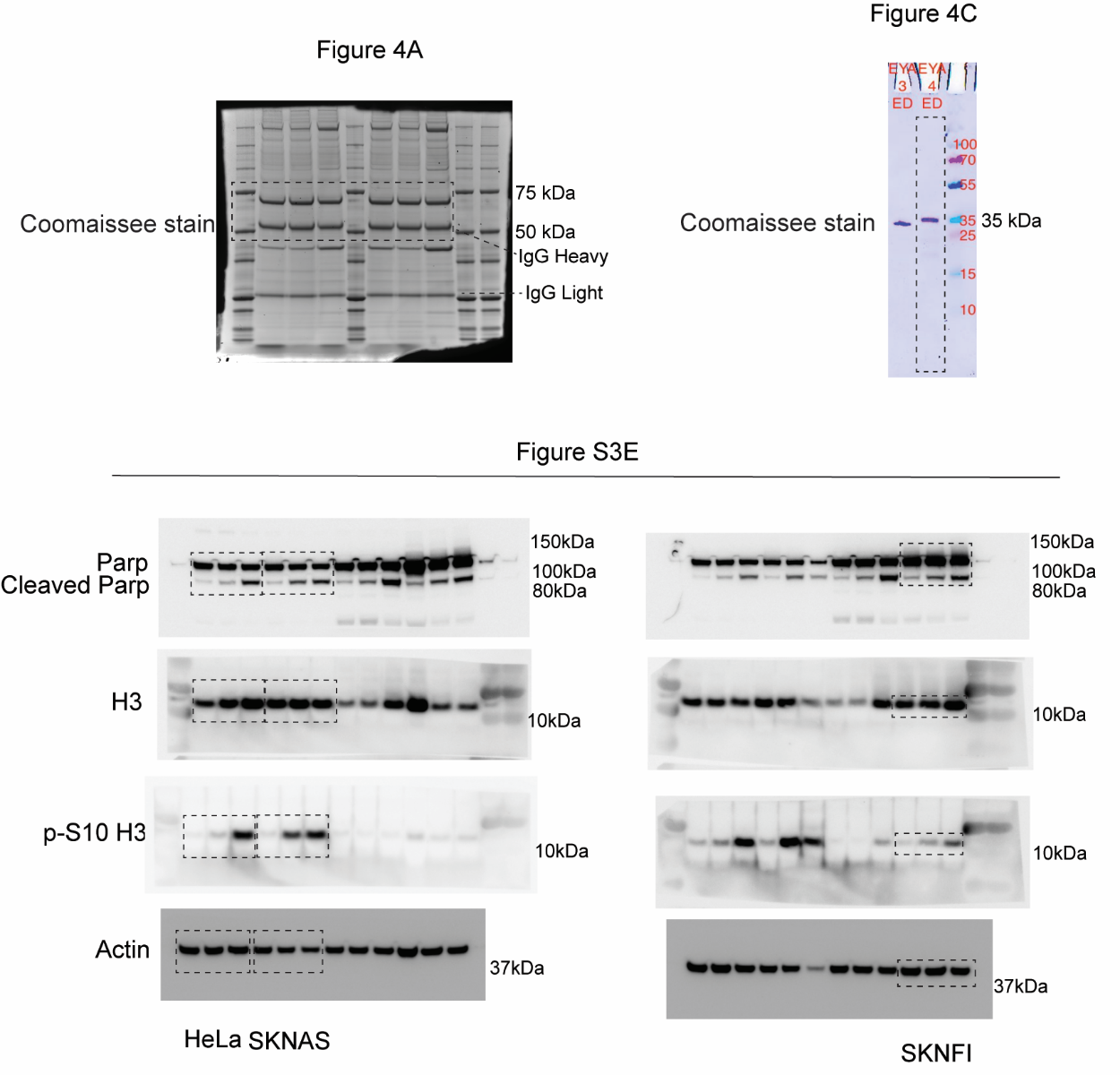


Figure 5 and Supplementary Figure 5:


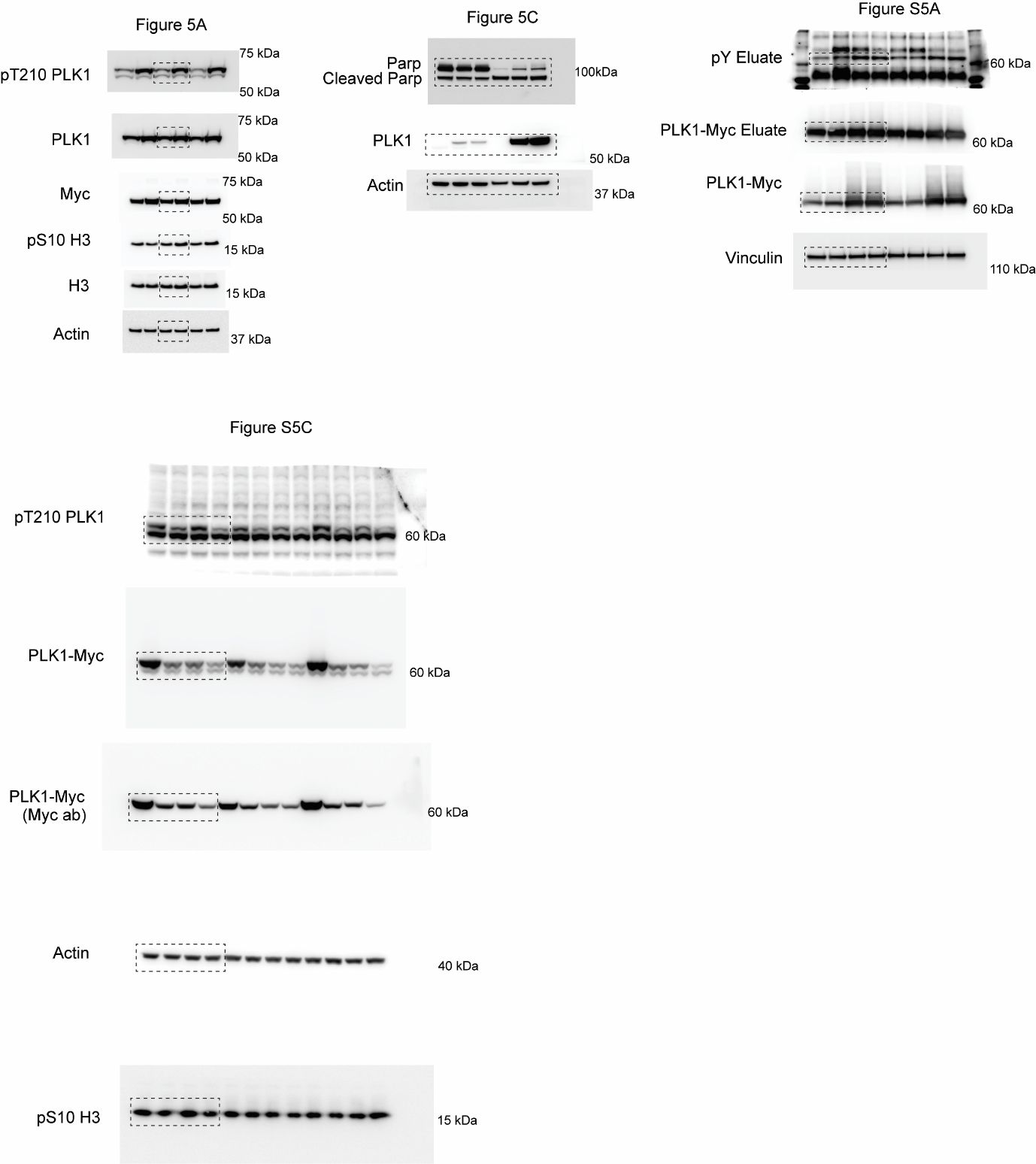


Figure 6:


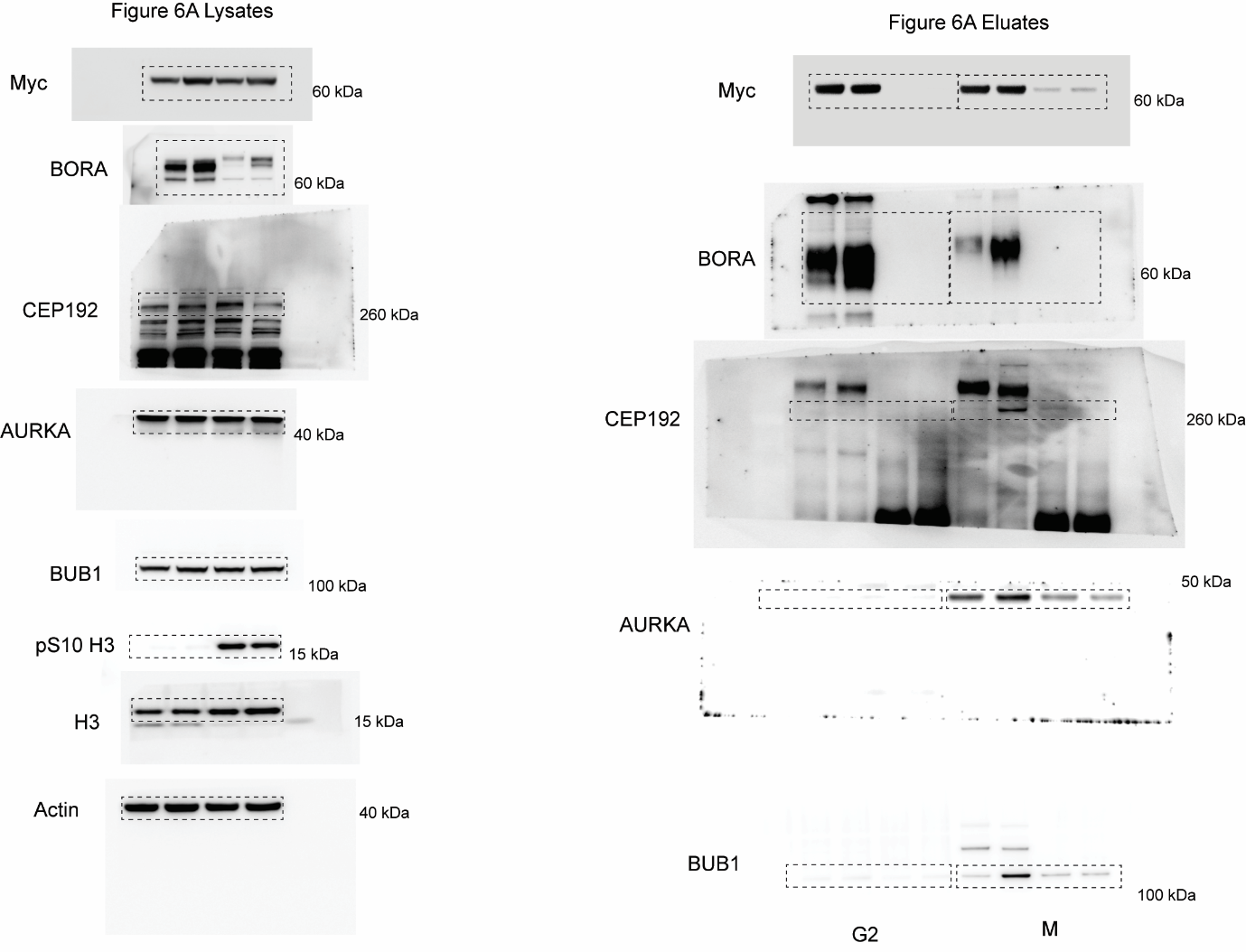

Supplement: Supplementary file 7 — Source Data [file 41467_2024_45683_MOESM7_ESM.zip › Sourcedata_WEsternblots.docx]
